# Improving the intrinsic thermal stability of the MAPbI_3_ perovskite by incorporating cesium 5-aminovaleric acetate†

**DOI:** 10.1039/c7ra13611k

**Published:** 2018-04-19

**Authors:** Xue Liu, Yulong Zhang, Jingchen Hua, Yong Peng, Fuzhi Huang, Jie Zhong, Wangnan Li, Zhiliang Ku, Yi-bing Cheng

**Affiliations:** State Key Laboratory of Advanced Technologies for Materials Synthesis and Processing, International School of Materials Science and Engineering, Wuhan University of Technology 122 Luoshi Road Wuhan Hubei P. R. China zhiliang.ku@whut.edu.cn; Hubei Key Laboratory of Low Dimensional Optoelectronic Material and Devices, Hubei University of Arts and Science 296 Longzhong Road Xiangyang Hubei Province P. R. China; Department of Materials Science and Engineering, Monash University Wellington Road Clayton VIC3800 Australia

## Abstract

Cesium 5-aminovaleric acetate (NH_2_C_4_H_8_COOCs) was used to improve the intrinsic thermal stability of the methylammonium lead triiodide (MAPbI_3_) perovskite. The corresponding carbon-based perovskite solar cells without encapsulation showed favourable stability at 100 °C for 500 h.

## Abstract 2

Cesium 5-aminovaleric acetate (NH_2_C_4_H_8_COOCs) was used to improve the intrinsic thermal stability of the methylammonium lead triiodide (MAPbI_3_) perovskite.

## Graphical abstract

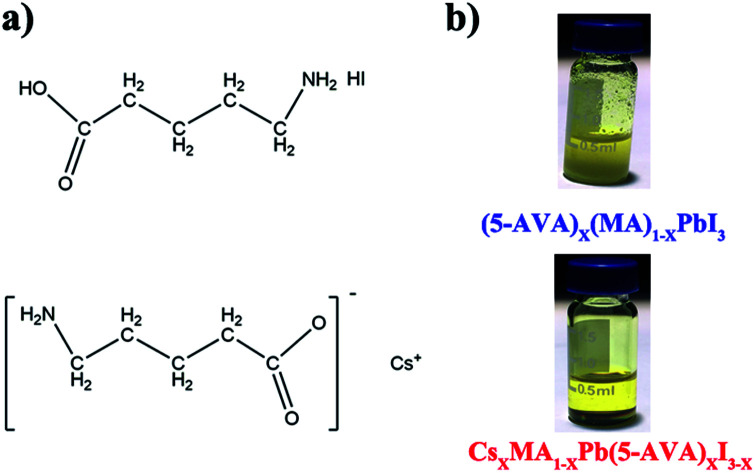

Next-generation solar cells have the prerequisite of low-cost and easy to fabricate are the prerequisites of next-generation printable solar cells. In recent years, printable solar cells based on ABX_3_ (where A is typically methylammonium (MA), formamidinium (FA), or Cs; B is Pb or Sn; and X is I, Br or Cl) perovskite-type light absorbers have attracted widespread attention for their high power conversion efficiencies (PCEs) combining with low processing costs. At present, the record PCE for any perovskite solar cell (PSC) is 22.7%,^1^ which is on a par with those of mainstream multi-crystalline silicon solar devices. Obviously, the high PCE together with easy fabrication procedures make PSCs a captivating device for future applications.

Typically PSCs need a vacuum system to evaporate a noble metal (Ag or Au) as the back contact. However, the use of vacuum system and expensive metal targets with high purity doesn't suit the purpose of low-cost printing. In this regard, the carbon-based perovskite solar cells,^2^ which substitute the noble metal electrode with a printable carbon electrode, have enormous potential for realizing the application of fully printed, low-cost photovoltaics. To date, many groups from all over the world have put much energy into the study of improving the performance of such PSCs with mesoporous carbon electrodes. Strategies including surface modification,^3–5^ materials engineering,^6–11^ solvent engineering^12–14^ and post-treatments^15–17^ have been applied in carbon-based PSCs for pursuing higher efficiencies. As a result, the reported PCE of carbon-based PSCs has increased rapidly from 6.6%^2^ to 17%^18^ in just a few years. And, at the same time, carbon-based PSCs have been reported that can be easily printed into large-scale modules.^19^ However, just like any other kind of PSC, long-term stability is still a problem for the final commercialization of this emerging photovoltaic technology.^20^ Solar modules are exposed to elevated temperatures during operation; hence as per the international standard (IEC 61646 climatic chamber tests), a solar panel must show thermal stability up to 85 °C.^21^ The formation energy for the MAPbI_3_ perovskite is 0.11–0.14 eV, which is close to 0.093 eV, suggesting possible degradation at a continuous exposure to a temperature of 85 °C. Recently, formamidinium cation (FA^+^)- and Cs^+^- based perovskites have demonstrated better thermal stability levels than has pure MAPbI_3_. However, FAPbI_3_ and CsPbI_3_ perovskites are more sensitive to humidity, which requires higher costs for encapsulation.^22,23^

In carbon-based PSCs, perovskite crystals are loaded in the mesopores, and their grain sizes are restricted by the pore size. In comparison to the perovskite crystals with large grains in traditional PSCs, perovskite crystals with small grains in carbon-based PSCs show much poorer heat tolerance. For this reason, all of the carbon-based PSCs using an MAPbI_3_ perovskite light absorber can only be annealed at 50–70 °C.^2,4,8,16,19^ From this perspective, enhancing the intrinsic heat tolerance of perovskite materials would be of great significance for the development of carbon-based PSCs.

Herein, we used cesium 5-aminovaleric acetate (NH_2_C_4_H_8_COOCs) as an additive of MAPbI_3_ perovskite, and found that Cs_*x*_MA_1−*x*_Pb(5-AVA)_*x*_I_3−*x*_ perovskite showed high heat tolerance, even at 150 °C. Based on previous work,^8^ 5-ammonium valeric acid (5-AVA) hydriodide (NH_2_C_4_H_8_COOH·HI), which was obtained from acid (HI) hydrolysis of 5-AVA, could create mixed-cation perovskite (5-AVA)_*x*_(MA)_1−*x*_PbI_3_ crystals with preferable stability in ambient air under full sunlight. Since 5-AVA is an amphipathic molecule, we employed CsOH to synthesize the alkaline hydrolysis product of 5-AVA (see ESI† for the synthesis details), aiming at combining the positive effects of the 5-AVA group and Cs^+^ cation in the MAPbI_3_ perovskite.

The molecular structures of (5-AVA) iodide and Cs-(5-AVA) acetate are shown in Fig. 1a. *Via* replacing 5% (in molar ratio) of MAI in the MAPbI_3_ perovskite with (5-AVA) iodide and Cs-(5-AVA) acetate, we obtained the (5-AVA)_*x*_(MA)_1−*x*_PbI_3_ perovskite and Cs_*x*_MA_1−*x*_Pb(5-AVA)_*x*_I_3−*x*_ perovskite, respectively. Note that 5-AVA served as a cation in the (5-AVA)_*x*_(MA)_1−*x*_PbI_3_ perovskite, while in the Cs_*x*_MA_1−*x*_Pb(5-AVA)_*x*_I_3−*x*_ perovskite, 5-AVA took the place of the I^−^ anion. This little difference resulted in different solubilities of the (5-AVA)_*x*_(MA)_1−*x*_PbI_3_ perovskite and Cs_*x*_MA_1−*x*_Pb(5-AVA)_*x*_I_3−*x*_ perovskite in γ-butyrolactone (GBL) solvent. As shown in Fig. 1b, the turbid (5-AVA)_*x*_(MA)_1−*x*_PbI_3_ perovskite solution (1 M in GBL) showed a lot of yellowish precipitate at room temperature. However, the Cs_*x*_MA_1−*x*_Pb(5-AVA)_*x*_I_3−*x*_ perovskite solution was much clearer, indicating the Cs_*x*_MA_1−*x*_Pb(5-AVA)_*x*_I_3−*x*_ perovskite to be more soluble than the (5-AVA)_*x*_(MA)_1−*x*_PbI_3_ perovskite. We attributed the good solubility of Cs_*x*_MA_1−*x*_Pb(5-AVA)_*x*_I_3−*x*_ perovskite in GBL to the exposed amino group of the Cs-(5-AVA) acetate.

**Fig. 1:**
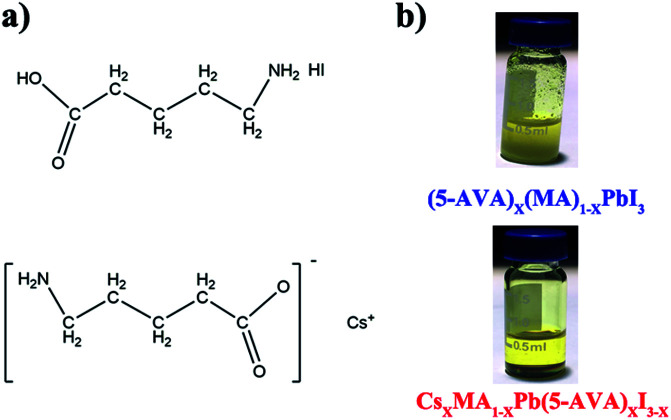
(a) The molecular structure of (5-AVA) iodide and Cs-(5-AVA) acetate. (b) Optical images of (5-AVA)_*x*_(MA)_1−*x*_PbI_3_ and Cs_*x*_MA_1−*x*_Pb(5-AVA)_*x*_I_3−*x*_ perovskite solution at room temperature.

X-ray diffraction (XRD) measurements were taken to identify the crystal structure of the as prepared perovskites. On glass substrate, both of the Cs_*x*_MA_1−*x*_Pb(5-AVA)_*x*_I_3−*x*_ and (5-AVA)_*x*_(MA)_1−*x*_PbI_3_ perovskite patterns showed similar diffraction peaks at 14.18°, 23.5°, 24.5° 28.56° and 31.05°, indicating the same tetragonal MAPbI_3_ perovskite crystal structure.^24^ What's more, the intensities of the Cs_*x*_MA_1−*x*_Pb(5-AVA)_*x*_I_3−*x*_ peaks were much stronger than those of the (5-AVA)_*x*_(MA)_1−*x*_PbI_3_ perovskite peaks. Since the two kinds of perovskite samples were fabricated and measured using the same conditions, we concluded the Cs-(5-AVA) acetate to be beneficial for the growth of the MAPbI_3_ perovskite (Fig. 2).

**Fig. 2:**
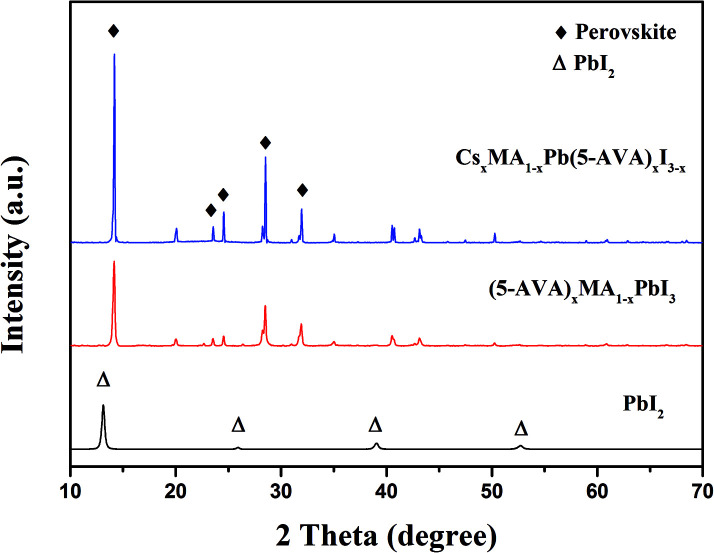
XRD patterns of the PbI_2_, (5-AVA)_*x*_(MA)_1−*x*_PbI_3_ and Cs_*x*_MA_1−*x*_Pb(5-AVA)_*x*_I_3−*x*_ films on glass substrates.

By using these two kinds of perovskite materials, we fabricated carbon-based PSCs according to the method reported previously.^2^ Briefly, by using the screen printing technique, mesoporous TiO_2_, ZrO_2_ and carbon films were successively deposited on the FTO substrates, which had been coated with compact TiO_2_ beforehand. And then, a perovskite light absorber was loaded by filling the precursor solution into the mesoporous carbon/ZrO_2_/TiO_2_ layers (see Fig. 3a). The microstructure of a cross-section of the as-prepared device was observed using SEM, and each layer showed well-defined boundaries and a uniform thickness (see Fig. 3b), and the distribution of the perovskite in the mesoporous films was homogenous (EDS images are shown in Fig. S1†). After the filling procedure, the devices were dried on a hot plate, and after the removal of GBL solvent, we finally obtained the carbon-based PSCs. For the sake of comparison, the drying temperature was set at 100 °C, which was much higher than the conventionally used temperature^8^ (50 °C).

**Fig. 3:**
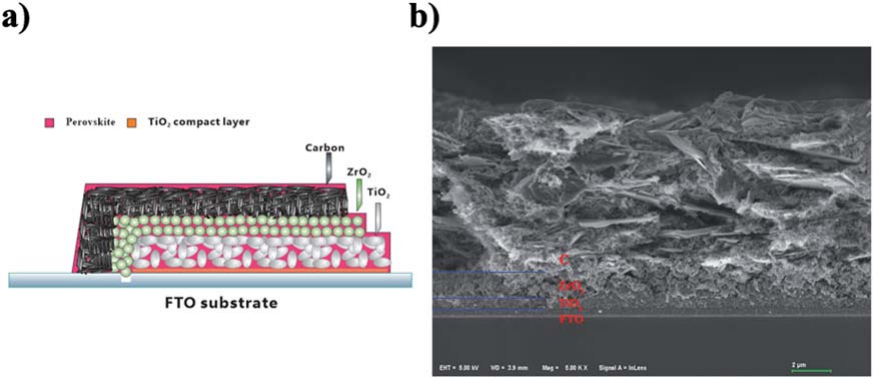
(a) Schematic structure of the carbon-based PSC. (b) SEM image from a cross-section of the carbon-based PSC.

Two groups of the carbon-based PSCs (with each group consisting of eight devices) were fabricated by using the Cs_*x*_MA_1−*x*_Pb(5-AVA)_*x*_I_3−*x*_ and (5-AVA)_*x*_(MA)_1−*x*_PbI_3_ perovskites, respectively. Under standard AM1.5 illumination (100 mW cm^−2^), the devices using the Cs_*x*_MA_1−*x*_Pb(5-AVA)_*x*_I_3−*x*_ perovskite generally showed a higher PCE than did the (5-AVA)_*x*_(MA)_1−*x*_PbI_3_ perovskite. (Details of the photovoltaic parameters are summarized in Table S1.†) *J*–*V* curves of the corresponding best devices are shown in Fig. 4a. With the Cs_*x*_MA_1−*x*_Pb(5-AVA)_*x*_I_3−*x*_ perovskite, the best device showed a photocurrent density (*J*_sc_) of 20.59 mAcm^−2^ (with *J*_sc_ determined from the IPCE spectra in Fig. S2†), an open circuit voltage (*V*_oc_) of 893 mV, a fill factor (FF) of 0.66 and an overall PCE of 12.19%. In contrast, the best device using the (5-AVA)_*x*_(MA)_1−*x*_PbI_3_ perovskite showed a much lower PCE of 9.50% (*J*_sc_ = 16.78 mAcm^−2^, *V*_oc_ = 830 mV and FF = 0.68). Moreover, the long-term stability levels of the best devices without encapsulation were determined at 100 °C in a glove box (see Fig. 4b). After 500 h, the PCE of the (5-AVA)_*x*_(MA)_1−*x*_PbI_3_-based device decayed gradually from 9.50% to 4.1%, *i.e.*, a reduction of 56.8%. Surprisingly, the Cs_*x*_MA_1−*x*_Pb(5-AVA)_*x*_I_3−*x*_-based device maintained 88% (decay from 12.19% to 10.73%) of its initial PCE after 500 h, indicating favourable thermal stability at 100 °C.

**Fig. 4:**
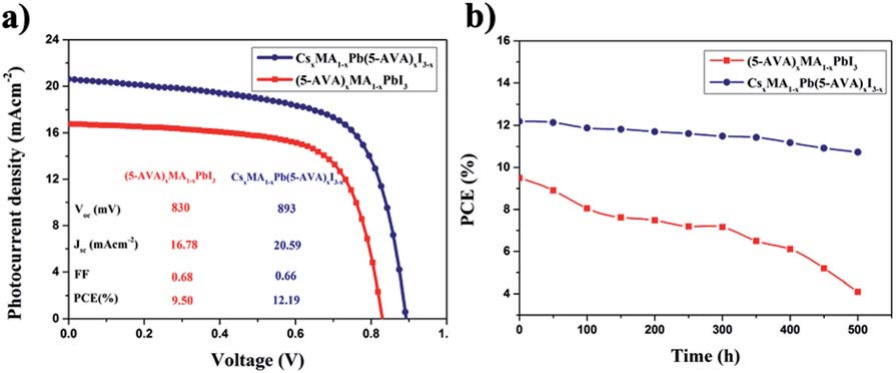
(a) *J*–*V* curves of the carbon-based PSCs with the (5-AVA)_*x*_(MA)_1−*x*_PbI_3_ and Cs_*x*_MA_1−*x*_Pb(5-AVA)_*x*_I_3−*x*_ perovskites. (b) Long-term stability of the carbon-based PSCs stored at 100 °C in glove box.

Further XRD measurements were taken to confirm the difference between the thermal stability of the (5-AVA)_*x*_(MA)_1−*x*_PbI_3_ perovskite and that of the Cs_*x*_MA_1−*x*_Pb(5-AVA)_*x*_I_3−*x*_ perovskite. The (5-AVA)_*x*_(MA)_1−*x*_PbI_3_ perovskite, after having been heated on a hot plate at 75 °C for 24 h, yielded an XRD pattern showing a small peak at 2*θ* = 12.63°, indicating that part of the perovskite was decomposed into PbI_2_. As the temperature was increased, the intensity of the PbI_2_ diffraction peak became increasingly stronger, and the colour of (5-AVA)_*x*_(MA)_1−*x*_PbI_3_ perovskite film on glass gradually changed from black to yellow (see Fig. 5a). However, compared with the (5-AVA)_*x*_(MA)_1−*x*_PbI_3_ perovskite, the Cs_*x*_MA_1−*x*_Pb(5-AVA)_*x*_I_3−*x*_ perovskite displayed a much lower thermal decomposition rate. As shown in Fig. 5b, even after having been heated at 150 °C for 24 h, most of the Cs_*x*_MA_1−*x*_Pb(5-AVA)_*x*_I_3−*x*_ perovskite still kept the perovskite phase (see Fig. 5b).

**Fig. 5:**
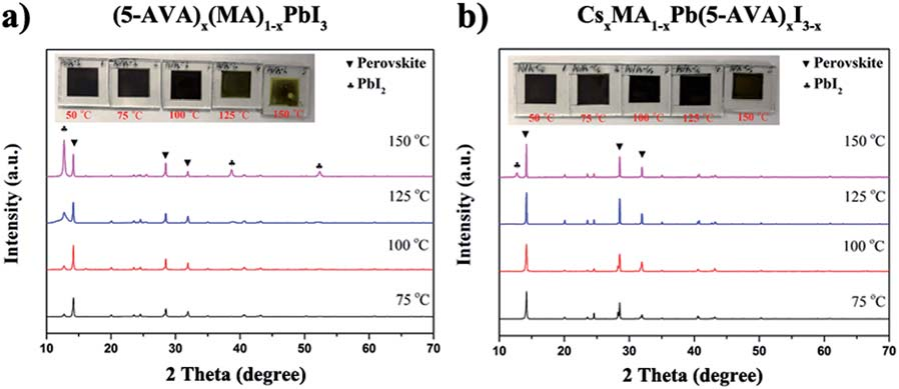
XRD patterns and optical images (inset) of (a) (5-AVA)_*x*_(MA)_1−*x*_PbI_3_ and (b) Cs_*x*_MA_1−*x*_Pb(5-AVA)_*x*_I_3−*x*_ perovskite films exposed on a hot plate to various temperatures each for 24 h.

To further identify any possible effects of the (5-AVA) anion and Cs cation on the thermal stability of the MAPbI_3_ perovskite, we also compared the decomposition process of MAPb(5-AVA)_*x*_I_3−*x*_ with that of Cs_*x*_MA_1−*x*_PbI_3−*x*_ (see Fig. S3†). The XRD results showed that including 5% Cs^+^ individually or 5% (5-AVA)^−^ individually in the MAPbI_3_ perovskite sample did not much enchance the thermal stability of the sample. Hence, we attributed the good thermal stability of the Cs_*x*_MA_1−*x*_Pb(5-AVA)_*x*_I_3−*x*_ perovskite to the unique Cs-(5-AVA) acetate additive. Since the 5-AVA group can act as a templating agent in perovskites^8,25,26^ and the Cs^+^ cation has a strong bonding energy with the I^−^ anion, the migration of ions in the Cs_*x*_MA_1−*x*_Pb(5-AVA)_*x*_I_3−*x*_ perovskite could be suppressed by the 2D/3D interfaces^27^ and the high migration activation energy of the inorganic cation.^28^

## Conclusions

In summary, we have developed a novel Cs_*x*_MA_1−*x*_Pb(5-AVA)_*x*_I_3−*x*_ perovskite by incorporating the Cs-(5-AVA) acetate into the MAPbI_3_ perovskite. Carbon-based PSCs using such Cs_*x*_MA_1−*x*_Pb(5-AVA)_*x*_I_3−*x*_ perovskites exhibited a favourable PCE of 12.19%. Moreover, after having been stored at 100 °C in a glove box for 500 h, the device still maintained 88% of its initial PCE. The XRD results showed the Cs_*x*_MA_1−*x*_Pb(5-AVA)_*x*_I_3−*x*_ perovskite to have a thermal decomposition rate much lower than that of the mixed-cation (5-AVA)_*x*_(MA)_1−*x*_PbI_3_ perovskite. Combining Cs^+^ with an alkyl chain to suppress the ion migration in MAPbI_3_ perovskites could be a new strategy for enhancing the thermal stability of PSCs.

## Conflicts of interest

There are no conflicts to declare.

## Supplementary Material

RA-008-C7RA13611K-s001
