# Supplementary material for: Improving the intrinsic thermal stability of the MAPbI3 perovskite by incorporating cesium 5-aminovaleric acetate
Source: RSC Adv. 2018 Apr 19;8(27):14991–4. doi: 10.1039/c7ra13611k (PMC9079981; doi:10.1039/c7ra13611k)
Supplement: RA-008-C7RA13611K-s001 [file RA-008-C7RA13611K-s001.pdf]

Electronic Supplementary Material (ESI) for RSC Advances.
This journal is © The Royal Society of Chemistry 2018

Supplementary Information

Experimental Section

Material: Cs-(5-AVA) acetate was synthesized by dissolving 1M 5-AVA (aladdin) in 1 M CsOH (50% wt in water) solution and then stirring for 2 h. Afterwards, via rotary evaporation, we obtained the Cs-(5-AVA) acetate and kept it in dry box for use. The $Cs_xMA_{1-x}Pb(5\text{-}AVA)_xI_{3-x}$ perovskite precursor was prepared by dissolving 578mg PbI_2, 187mg MAI and 15.56mg Cs-(5-AVA) acetate in 1mL γ-butyrolactone (GBL) under room temperature. The $(5\text{-}AVA)_x(MA)_{1-x}PbI3$ perovskite precursor was prepared as the recipe reported previously[1].

Device Fabrication: The FTO glass (Kaivo TEC 15, 15 Ω sq^{-1}) that has been etched with a laser to form two detached electrode patterns, and then ultrasonically cleaned with detergent, deionized water, and ethanol. The TiO_2 dense layer was deposited on the FTO glass by spray pyrolysis deposition with a solution involving the titanium di-isopropoxidebis (acetylacetonate) diluted in ethanol (1:9, v/v) under 450 ^oC. TiO_2 mesoscopic layer was fabricated via screen printing with TiO_2 paste (DSL 30NR-T, Dyesol, Australia, diluted with terpinolby weight ratio 1:2.25), followed with sintering under 500 ^oC; ZrO_2 and carbon layer were printed with corresponding paste, followed with sintering under 500 ^oC and 400 ^oC, respectively. The as-prepared mesoscopic films were infiltrated with perovskite precursor solution via drop coating on top of the carbon layer. Then, the devices were dried at 100 ^oC for 1 h. After the removal of the solvent, we finally obtained the carbon-based PSCs.

Characterization: The XRD spectra were measured with a Bruker D8 Advance X-ray diffractometer with Cu Kα radiation (λ=1.5418) from 10° to 80°. The morphological characterizations were performed by field-emission scanning electron microscopy (FE-SEM, Zeiss Ultra Plus) equipped with an EDS detector. Photocurrent-voltage (J-V) characteristics were conducted by a solar simulator (Oriel 94023A, 300 W) under standard light intensity of AM1.5 and $100mWcm^{-2}$ using a non-refractive mask with an aperture area of 0.1475 cm^2 at a scan rate of 0.1 V/s.

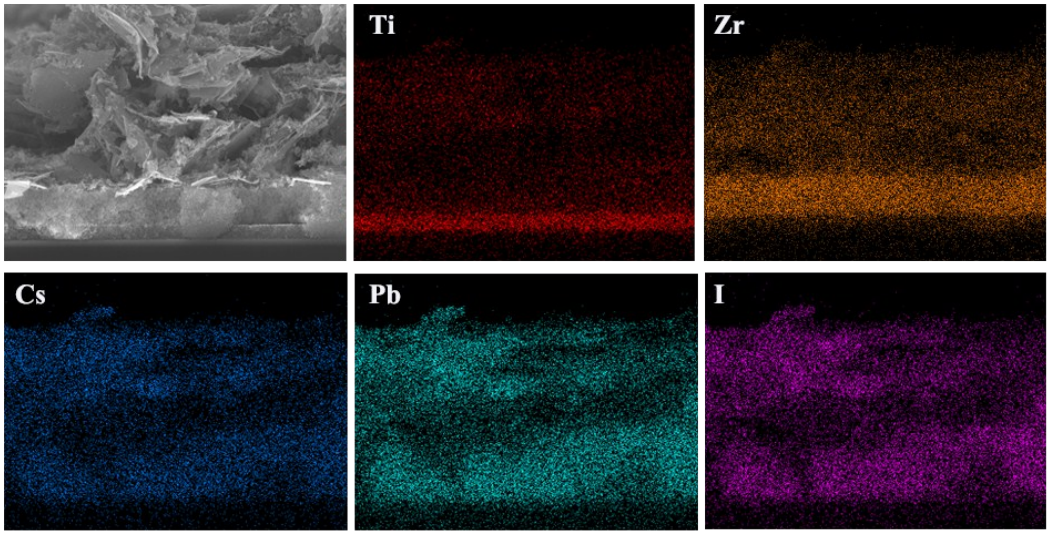

Fig. S1 EDS mapping of the carbon-based PSCs form cross-section.

From the EDS mapping, Cs, Pb and I element are distribute homogenously in the TiO_2/ZrO_2/Carbon triple layers, indicating the full-filling of $Cs_xMA_{1-x}Pb(5\text{-}AVA)_xI_{3-x}$ perovskite $(5\text{-}AVA)_x(MA)_{1-x}PbI_3$ perovskite in carbon-based PSCs.

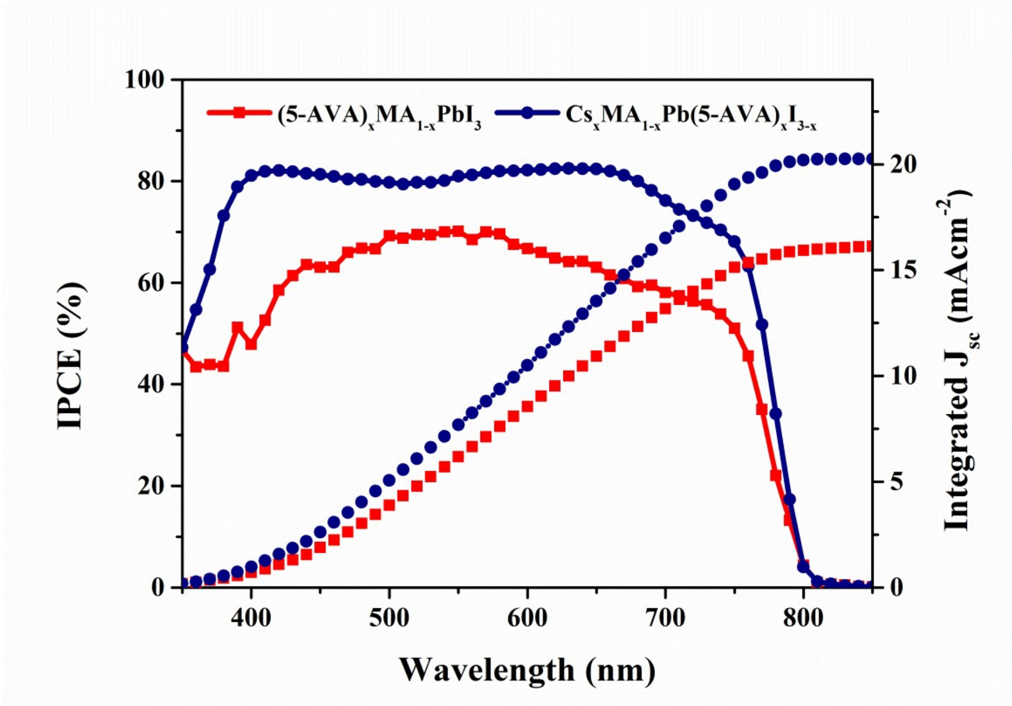

Fig. S2 IPCE spectra of the champion cells with $(5\text{-}AVA)_x(MA)_{1-x}PbI_3$ perovskite and $Cs_xMA_{1-x}Pb(5\text{-}AVA)_xI_{3-x}$ perovskite.

The photocurrent density of the champion devices were checked by IPCE spectra. As shown in figure S1, the $Cs_xMA_{1-x}Pb(5\text{-}AVA)_xI_{3-x}$ perovskite based cell generates a photocurrent up to 800 nm, which is in accordance with the absorption range of the $MAPbI_3$ perovskite. The integration of the IPCE spectra of the champion device with $Cs_xMA_{1-x}Pb(5\text{-}AVA)_xI_{3-x}$ perovskite gives 20.25 $mAcm^{-2}$, which is in rather good agreement with the J_{sc} obtained from the J-V curve (20.59 $mAcm^{-2}$).

Table. S1 Photovoltaic parameters of the carbon-based PSCs with different perovskite.

Device	No.	Voc (mV)	Jsc ($mAcm^{-2}$)	FF	PCE (%)
	1	909	12.85	0.681	7.96
	2	830	16.78	0.682	9.50
	3	841	17.41	0.638	9.33
	4	816	16.44	0.597	8.01
**$(5\text{-}AVA)_x(MA)_{1-x}PbI_3$**	5	891	16.44	0.592	8.68
	6	872	15.65	0.592	8.08
	7	835	15.54	0.636	8.25
	8	838	16.40	0.631	8.67
	**Average**	854	15.94	0.631	8.56
	1	896	21.97	0.604	11.88
	2	890	21.44	0.594	11.34
	3	881	21.90	0.598	11.54
	4	923	21.13	0.602	11.74
**$Cs_x(MA)_{1-x}PbI_{3-x}(5\text{-}AVA)_x$**	5	910	19.24	0.659	11.53
	6	893	20.59	0.663	12.19
	7	885	19.05	0.649	10.94
	8	875	19.26	0.694	11.69
	**Average**	894	20.57	0.633	11.61

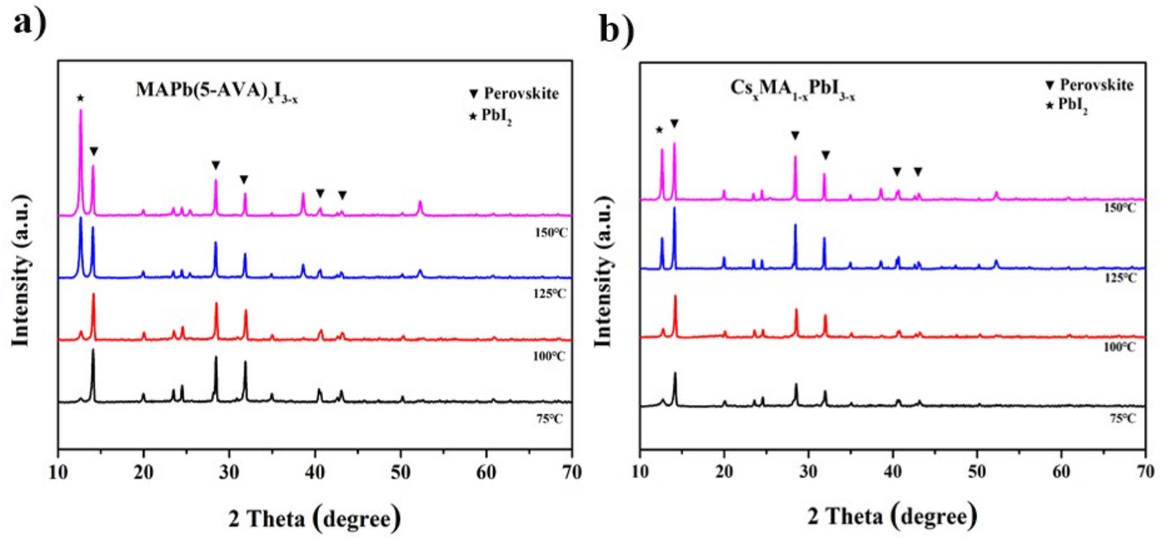

Fig. S3 XRD patterns of $MAPb(5\text{-}AVA)_xI_{3-x}$ and $Cs_xMA_{1-x}PbI_{3-x}$ perovskite after heated at different temperature.

As shown in figure S3, both of $MAPb(5\text{-}AVA)_xI_{3-x}$ and $Cs_xMA_{1-x}PbI_{3-x}$ perovskite exhibit similar decomposition rate with $(5\text{-}AVA)_xMA_{1-x}PbI_3$ perovskite at high temperature. Hence, we contribute the stability of $Cs_xMA_{1-x}Pb(5\text{-}AVA)_xI_{3-x}$ perovskite to the combined effect of Cs^+ and $(5\text{-}AVA)^-$.

1. A. Mei, X. Li, L. Liu, Z. Ku, T. Liu, Y. Rong, M. Xu, M. Hu, J. Chen, Y. Yang, M. Gratzel and H. Han, *Science*, 2014, **345**, 295-298.
